# A qualitative study on resilience in adult refugees in Germany

**DOI:** 10.1186/s12889-021-10817-6

**Published:** 2021-04-30

**Authors:** Lena Walther, Julia Amann, Uwe Flick, Thi Minh Tam Ta, Malek Bajbouj, Eric Hahn

**Affiliations:** 1 Department of Psychiatry and Psychotherapy, Charité University Medicine Berlin, Berlin, Germany; 2 Department of Education and Psychology, Freie Universität Berlin, Berlin, Germany

**Keywords:** Resilience, Refugees, Asylum-seekers, refugee mental health, Integration

## Abstract

**Background:**

Because refugees face significant adversities before, during, and after resettlement, resilience is of central importance to this population. However, strengths-based research on post-migration refugee experiences is sparse.

**Methods:**

We conducted semi-structured interviews with 54 adult refugee participants who arrived in Germany between 2013 and 2018 in their preferred language. We analyzed different aspects of resilience in these interviews using thematic analysis.

**Results:**

Nine themes were identified. Four themes manifest resilience in different ways and encompass cognitive as well as behavioral strategies for facing adversity, self-ascriptions of resilience as a personal trait or lasting characteristic, and the role of volunteering, work, and activism for refugee causes. Five themes capture factors that facilitate resilience: social support, experiencing migration as an opportunity generally and for women in particular, being a parent, and being young.

**Conclusions:**

This study adds to a growing body of knowledge about resilience among adult refugees. It may support clinicians working with refugees by making them aware of specific manifestations of resilience and factors promoting positive adaptation specific to this client group. It also contributes to a more strengths-based view on refugee mental health and processes of integration.

**Supplementary Information:**

The online version contains supplementary material available at 10.1186/s12889-021-10817-6.

## Introduction

Refugees and asylum seekers (henceforth referred to as “refugees”) face a range of adversities prior to, during, and after migration. Following exposure to various types of violence, loss, and life-threatening circumstances in their country of origin and during flight, refugees experience a multitude of challenges in receiving countries. These include protracted periods of uncertainty regarding prospects of staying in the receiving country, struggles with learning a new language and joining the labor market, stretches of involuntary inactivity and boredom, social isolation and discrimination [1].

Perhaps not surprisingly, the focus of research on mental health among refugees has predominantly been on these adversities’ negative psychological sequelae, including depression, anxiety, and post-traumatic stress disorders. While mental health struggles are prevalent among refugees, it is also the case that a substantial proportion of this population does not appear to develop these psychological disorders [2] and that many find ways to rebuild and thrive [3]. In fact, it is plausible that self-selection mechanisms are at work whereby individuals with particular strengths and resources are more likely to risk flight and become refugees in the first place (e.g. positive selection on the level of education among Syrian and Iraqi refugees in Germany, [4]). The need for research and clinical practice to incorporate a strengths- rather than deficits-based view on refugee experiences in order to reflect this reality and avoid pathologization has been increasingly recognized [5–9]. Strengths-based views, both in clinical practice (e.g. [10]) and in academic research (e.g. [11]), essentially revolve around the key concept of resilience and have the ultimate aim of promoting resilience.

In line with a seminal work from developmental psychology, we consider resilience to be the “process of, capacity for, or outcome of successful adaptation despite challenging or threatening circumstances” ([12], p. 426). “Successful adaptation” or “positive adaption”, as it is more commonly called, encompasses mental health and well-being as well as functioning [13]. Depending on the nature and severity of the adversity, different levels of mental well-being and functioning may constitute positive adaptation [14, 15]. A further concept closely related to resilience is coping, defined as cognitive or behavioral efforts to manage external or internal stressors ([16], p. 141, cited in [17]). We follow Rice and Liu [17] in considering coping mechanisms to be part of the process of adaptation to adversity. Additionally, factors that promote successful adaptation, such as an individual’s characteristics and resources, are of particular interest in the study of resilience [14, 15, 18].

Importantly, this is a psychological perspective on resilience, as opposed to one that also addresses resilience on a social environment level (e.g. [19]). Ungar et al. [19], for example, include within their understanding of resilience “not only an individual’s capacity to overcome adversity, but the capacity of the individual’s environment to provide access to health-enhancing resources” (pg. 288). Without any intention to de-politicize the topic of refugee well-being [20], the present study focuses on psychological resilience in order to inform clinical practice and our understanding at an individual psychological level.

Research on psychological resilience within refugee experiences has the potential to be of great value to clinicians working with this population, making them aware of potential sources of strength specific to this client group [6, 7]. Because the process of integration produces many challenges and also requires high levels of functioning [21], resilience is also of paramount importance to integration. However, relatively few studies have focused on resilience among refugees – particularly adult refugees [9], and fewer still take a qualitative approach. Qualitative approaches are especially well-suited for uncovering new factors related to understudied phenomena and understanding lived experience in greater detail [22–24].

Existing qualitative research addressing resilience or coping among adult refugees has repeatedly identified social support as vital for the achievement and maintenance of well-being in the face of adversity [11, 25–32]. Cognitive strategies such as positive attitudes and beliefs, appraisals and self-talk centering around affirmations of inner strength, agency, hope, and optimism are another major resilience-related aspect highlighted across studies [11, 26, 28, 29, 32, 33]. A third recurring theme linked to resilience in refugee samples is religion and spirituality [25, 26, 28, 30, 32]. These studies have provided valuable insights into resilience among refugees, demonstrating the potential of qualitative research for this growing research area. It is of note, however, that most of these studies cover refugees’ experiences more broadly and consequently feature rather brief explorations of factors related to resilience (e.g. [28, 29, 31–33]; an exception, e.g.: [11]). Relatively few qualitative studies focusing on resilience in adult refugees have been conducted in Germany, one of the major receiving countries for refugees in recent years [34]. Between 2013 and 2018, Germany's refugee population increased by 1.2 million – mostly from Syria, Afghanistan, Iraq, Iran, Pakistan, Eritrea, Somalia, and Nigeria [3].

The present study takes an in-depth look at different aspects of psychological resilience in 54 adult refugees who arrived in Germany between 2013 and 2018 based on the analysis of semi-structured, qualitative interviews. More specifically, the study addresses ways in which the process of, capacity for, and the outcome of successful adaptation to adversity manifest, as well as factors facilitating successful adaptation.

## Methods

### Participants and sampling

Our sample comprised 54 adult participants who arrived in Germany between 2013 and 2018 through forced migration (self-reported). Most participants arrived in 2015, the year that saw the largest number of new arrivals to Germany by far [3]. Participants resided in Berlin, Berlin; Leipzig, Saxony; or the Duisburg area – mainly the city Mülheim an der Ruhr (two interviewees from Duisburg, one from Dinslaken), North Rhine-Westphalia, Germany, at the time of the interviews. We recruited from three different areas in case we might find significant particularities in experiences based on place of residence. Only four interviews were conducted in Leipzig due to logistical constraints. Recruitment strategies included outreach on social media and through refugee organizations (Additional file 1 includes study flyer text) as well as snowballing. We increased our selectivity concerning the age, gender, education background, and country of origin of participants in the recruitment process to achieve greater variation. In particular, as recruitment progressed, we increased our efforts to recruit participants from Afghanistan, female participants, older participants, and participants with limited educational backgrounds. Recruitment continued until we reached our sampling goals: diversity along the demographic factors mentioned, as well as approximate gender parity, substantial numbers of participants from both of the main countries of origin – Syria and Afghanistan.

### Topic guide

The topic guide (included in the Additional file 1) was designed following guidelines on good practice in semi-structured interviews (e.g. [35, 36]) and input from our Arabic and Farsi/Dari native language interviewers, one of whom is a member of the exact community under study and two others who are first and second-generation migrants from countries represented in our sample. It encompassed three sections: first, a personal background section; second, a section on cultural experiences; and third, a section on emotions, well-being and mental health. We trialed a partial version of the topic guide in eight pilot interviews not used in the present study. Based on the first complete interviews included in this study, a few questions were added to the topic guide because interviews turned out shorter than expected, allowing for further questions. Also, one participant among the first struggled to open up about their personal situation, prompting us to add more general questions (e.g. “Do you think refugees in general face mental health challenges/ emotional stress? Why yes/no?”). Please see the topic guide in the Additional file 1 for questions flagged as “added after first interviews.”

### Data collection

Data collection took place between December 2018 and September 2019 in Berlin, Leipzig, and Mülheim an der Ruhr. Interviews were conducted in person in locations chosen by participants (usually cafés), semi-structured, on a single occasion, typically one-on-one (four interviews with translator and six with a family member of participants present, with one interview of two brothers analyzed as two separate interviews because both brothers answered our questions individually), and audio-recorded in all but one case. In this one case, the interviewer took notes which we used as data on topics raised but were not able to include details from. Durations ranged from approximately 30 to 90 min. Participants were not financially compensated for their participation; refreshments were paid for.

Participants chose their preferred interview language in advance (22 Arabic-, 10 Farsi/Dari-, 19 German-, and 3 English-language interviews) and matched with one of our seven interviewers based on this preference, as well as on the basis of interviewer availability. In the final phase of the study, we were only able to interview individuals able to speak German or English due to study logistics; this affected 10 interviews. Interviewers, who were all provided with interview technique guides, including ethics instructions, and a brief on study aims, included: a female Syrian-American Arabic-speaker (anthropologist), a male Syrian Arabic-speaker (sociologist), a male Iranian Farsi-speaker (professional psychiatric translator), and four German German- and English-speaking interviewers (psychologists and sociologists, including LW and JA).

The interviewers and external professional translators transcribed and translated the voice recordings into English or German. The quality of these translations was checked and confirmed by other professional translators based on a random selection of one interview from each translator.

### Data analysis

We analyzed our data using the thematic analysis approach presented in Braun and Clarke’s [37] seminal methodological framework. Following data immersion in the form of reading all transcripts, LW and JA independently applied, discussed, and amended open but detailed codes line-by-line to the well-being section of the transcripts as well as to segments related to well-being and adaptation in the first sections using MAXQDA. We also created an overview table summarizing participants’ stories, including summarizing observations on their mental health, well-being, and adaptation to their new surroundings in order to make it easier to maintain a complete within-case understanding of interview contents throughout the analysis process [38]. Next, we analyzed codes and corresponding interview passages that were linked, broadly, to “overcoming or facing adversities”, including passages about facing hardships, about positive well-being and functioning, and other displays of strength, arriving at themes in an iterative process of categorization, discussion, and categorization. Within this process, we also noted associations between participants’ characteristics and facing adversity that we observed. We created code maps using MAXQDA’s visualization tools to assist this analysis process.

Beyond setting a focus on overcoming adversities, we approached the data largely inductively. We did not formulate specific resilience-related categories based on the literature a priori, although our familiarity with the concepts “coping strategies” and “social support” played into our thematic categorizations early on. Eventually, the theoretical literature on resilience guided our understanding of how the themes we identified figured into resilience. Due to the succinctness of the definition and its openness with regard to whether resilience is a process, a capacity, or an outcome, we specified our concept of resilience as “process of, capacity for, or outcome of successful adaptation despite challenging or threatening circumstances” ([12], p. 426). We identified some of our themes as manifesting resilience in these different forms. We identified the rest of our themes as representing factors that facilitate resilience understood in this way.

## Results

Table 1 shows the sample characteristics.

**Table 1:** Sample Characteristics

***Gender***	**Female**	**Male**					
	24	30					
***Age***	**18–24**	**25–29**	**30–34**	**35–39**	**40–44**	**45–49**	**50–55**
	11	13	12	5	5	3	5
***Country of origin***	**Syria**	**Afghanistan;Afghanistan/Iran**	**Iran**	**Pakistan**	**Palestine**	**Libya**	**Sudan**
	36	9	4	2	1	1	1
***Level of Education***	**No secondaryeducation**	**Secondaryeducation**	**Started university in the country of origin**	**University-educated**	**Young and currently in secondary education**	**Unknown**	
	5	3	9	28	3	6	
***Residence in Germany***	**Berlin, Berlin**	**Leipzig, Saxony**	**Mülheim an der Ruhr, Duisburg, or Dinslaken, North Rhine-Westphalia**				
	39	4	11				
***Year of arrival in Germany***	**2013**	**2014**	**2015**	**2016**	**2017**	**2018**	
	1	1	34	11	6	1	
***Legal status***	**Refugee or asylum status**	**Subsidiary protection or deportation ban**	**Unresolved**	**Humanitarian program**	**Family reunification**	**Visa sponsorship**	**Unknown**
	25	10	11	1	3	1	3
***Housing***	**Private**	**Housing facility**	**Unknown**				
	30	15	9				
***Occupation***	**Gainfully employed**	**In education**	**None reported**				
	13	11	30				

*Gender, age, country of origin, residence in Germany, year of arrival in Germany, legal status, and housing situation were directly ascertained (although housing situation and legal status were sometimes not entirely clear or unclear). Level of education and occupation were interpreted based on interview content. The "Unresolved" legal status category includes those waiting for the outcome of their appeal*

Table 2 shows the nine themes we identified, specific points within each, as well as the two broad categories into which we organized themes based on their function within resilience. The themes *Cognitive coping strategies, Behavioral coping strategies, Self-ascribed resilience as an enduring capacity,* and *Volunteering, activism, and work for refugee causes* all capture ways in which participants manifest resilience as the “process of, capacity for, or outcome of successful adaptation despite challenging or threatening circumstances” ([12], p. 426). The themes *Social support, Experiencing migration as an opportunity for self-expression, belonging, and personal development, Experiencing migration as an opportunity for women, Being a parent,* and *Being young* all cover factors that appear to facilitate successful adaptation. Within overarching categories, themes are in no particular order except that *Cognitive and Behavioral coping strategies* and *Social support,* the first themes in the two categories, were the most globally represented among our participants. The sections below include interview quotations with the individuals quoted represented as “P” for “participant” and a participant number, e.g. “P1”.

**Table 2:** Thematic Map

Themes’ Function within Resilience	Themes	Theme Contents
**Manifestations of process of, capacity for, and outcome of successful adaptation**	**Cognitive coping strategies**	• Acceptance • Focus on the present or future • Active forgetting • Focus on daily tasks • Belief in an internal locus of control • Favorable comparisons between life in Germany and life in the country of origin • Comparisons to peers • Growth through adversity mindset
	**Behavioral coping strategies**	• Work as distraction • Withdrawal from stressors • Connecting to cultural roots or faith • Processing through creative outlets • Seeking mental health care
	**Self-ascribed resilience as an enduring capacity**	• Character traits • Learned, life-long positive attitude • Resilience due to good past
	**Volunteering, activism, and work for refugee causes**	• Being active for refugee causes as a manifestation of psychological and other resources, sense of one’s rights • Giving meaning to hardships, distance from hardships, agency and identity, sense of community • Using strengths to be a voice for peers • Activism in the country of origin turned activism in the host country; proactive individuals
**Factors facilitating successful adaptation**	**Social support**	• Acceptance, feeling more at ease, sense of belonging, concrete support • Infrastructure for social support (tandems, meet-ups) can be vital • Language teachers as important contacts • Family
	**Experiencing migration as an opportunity for self-expression, belonging, and personal development**	• Living more in keeping with values post-migration • Enjoying greater freedom • Opportunity for learning and personal development • Having wanted to migrate • Appreciation of multiculturalism and diversity
	**Experiencing migration as an opportunity for women**	• Opportunities for women beyond motherhood • Freedom to choose and pursue education and career • Feelings of youthfulness due to opportunities • Changes in the marital relationship
	**Being a parent**	• Children’s opportunities • Remaining hopeful for children • Reducing stress for children • Meaning from children • Children as a new beginning
	**Being young**	• Not such severe loss of status • Friends at the educational facility as key to well-being • Clear metrics of success within an educational facility • Educational facilities as suitable contexts for integration

## Manifestations of process of, capacity for, and outcome of successful adaptation

### Cognitive coping strategies

Our participants exhibited several different cognitive strategies for overcoming or adaptively reappraising the adversities they face. These strategies manifest aspects of the process of as well as the capacity for successful adaptation. They include acceptance, focus on present or future, belief in an internal locus of control, favorable comparisons between life in Germany and life in the country of origin, comparisons to peers, and growth through adversity mindset.

An **acceptance** of circumstances and challenges – including the whole situation of forced migration (quote 1 below), uncertainty about the future (quote 2), and slow progress in the integration process (quote 3) – allows some participants to focus on building their lives in Germany. Sometimes this acceptance appears to be the outcome of a long phase of disappointment over how much more difficult post-flight life is than previously expected.

^1^ “*These are our circumstances. We are in this situation. We have to accept the situation and find a way to deal with this new life. Otherwise, we will become nothing*.” (P51).

^2^ “*I don’t worry at all about whether I will be allowed to stay in Germany – that was only at the beginning. I think if things were meant to happen, then they will happen*.” (P53).

^3^ “*In Germany, we have a normal kind of stress as newcomers in the process of migration. You have to go this path [ …*]. *It’s a natural stress that you have to accept.*” (P38).

Another form of adaptive thinking in our participants is a **focus on the present or the future**. It often entails an acceptance of past experiences and losses, as is made explicit in the first quote below. Hope for the future, expressed as confidence in being able to “build something” (quote 1), and curiosity regarding things to come (quote 2) are further potential facets of this attitude.

^1^ “*I am building something here. Even though it’s little steps, I prefer that compared to looking back at what I’ve lost and crying over it.*” (P49).

^2^ “*I’m someone who wants to experience a lot of new things. I want to see new places [ …*], *get to know new people and cultures, languages, I just love that. I think that’s where my strength comes from.*” (P50).

In addition to focusing on the present or the future, some of our participants make an effort to **actively forget past adverse experiences** to protect their mental health. Implementing this strategy is an ongoing challenge. The first quote below shows how intentional some are about actively forgetting. The second quote is from a participant who says he learned to suppress negative experiences as a form of adaptation whilst caring for the wounded during war.

^1^ “*I want to be honest about my experiences [in this interview]. At the same time, I cannot explain everything in detail because I am trying to forget things that have happened to me in order to lead a new life.*” (P10).

^2^ “*[ …*] *And with time, I changed. I don’t think about the bad things that happen to me [anymore].*” (P46).

Some employ a **focus on daily tasks** as a means to confront paralyzing feelings of uncertainty and doubt. While many participants described feeling demotivated due to not knowing whether their efforts will bear fruit, the first quoted below frames his daily activities as “duties” to avoid a need for confidence in future prospects. Another participant repeatedly mentioned the importance of using a planner and filling it with appointments, saying that staying busy keeps him content and begets motivation (quote 2).

^1^ “*I have a duty to do. I am registered as a refugee; they told me I have to go to course, then I go to course, I learn German, I finish my German and then I find a job. I don’t think about what’s gonna come next. I stop worrying about whether they’re gonna send me, not send me back [ …]. I just pursue my daily life.*” (P48).

^2^ “*When we started the language courses, we got motivated and then kept posted with events going on with Facebook’s help, then I started using a calendar just like Germans and writing down my appointments [ …] and all that gives you a motivation because you always have a new thing to do [ …], I like to keep busy.*” (P12).

Some participants invoke an **internal locus of control** and report trying – albeit with difficulties – to focus on their personal sphere of influence to try to self-activate (quotes 1–2 below). These statements stand in juxtaposition to many expressions of feeling at the mercy of circumstances and hindered by various restrictions in our interviews. In an utterance directly addressing the struggle to secure a sense of agency in the face of the overwhelming external forces refugees experience, one participant distinguishes areas under external from areas under internal control and focuses on the latter (quote 2).

^1^ “*[ …] you have to take charge of yourself. My energy comes from the fact that after three years, I will be asked what did you do in Germany. [ …] And the answer is more important to me than to them. Yeah, what did I do? I got the B2 certificate, I am doing a traineeship [ …]. This is something; it’s good for me. [ …] You have to think about yourself.*” (P48).

^2^ “*It was not my choice to have been born in [country] and to be [trait], but it was my choice to be saved, and that’s why I only think about having been saved.*” (P38).

Participants reappraised their present struggles through **favorable comparisons between life in Germany and life in the country of origin** (quotes 1–3 below). A focus on the appreciation of personal safety is often central to this attitude. Some also frame their time in Germany as a unique opportunity to develop in ways not possible in the country of origin (quote 2). In contrast to the many participants who find contact with administrative bodies stressful or even demeaning, one participant makes comparisons to the country of origin to frame these interactions (quote 3).

^1^ “*When encountering a bad situation here, one will always remember how bad it was in Syria. Here one will feel like heaven because the stressors in Syria and the psychological wars were horrible.*” (P17).

^2^ “*In my country, people are experiencing harsh living conditions, here [in Germany] life is good. I should use this chance - of me being here - so I can build something. There, in my country, we cannot build anything.*” (P37).

^*3*^ “*I tell my Syrian friends when complaining, ‘Please remember how we are treated in Syria then.*

*compare that to here and you will see the difference’. Some feel annoyed by the Job center appointments, but why? […] [In] our country, [there is] bribery and corruption, so when I am treated with respect here, I feel happy to be here.*” (P27).

Our participants develop their own attitudes towards the challenges they face through **comparisons with their peers**. The first quote below shows how some remind themselves that they have it easier than their peers because of family support, their language skills, or their educational background. Quotes 2 and 3 expresses how some are motivated by hearing about the successes or struggles of others.

^1^ “*For me, it’s very important to understand what people are suffering from so I can appreciate how lucky I am.*” (P48).

^2^ “*I was speaking to a friend who came by sea a month after me, he said that he got C1 degree in German language and at that moment I woke up! Like what was I doing with my time!*” (P19).

^3^ “*It’s like a fine line between you and giving up, as a refugee. You know many people give up. [ …] And that might happen to me, so how am I going to face it? By keeping on doing things.*” (P48).

Several participants exhibit a **growth through adversity mindset**, conceiving themselves as empowered by the hardships they have overcome (e.g. quotes 1–2 below). One participant contrasts his and his fellow Syrians’ hardiness as a consequence of this growth with the overreactions to problems he observes among Germans (quote 2).

^1^ “*The past that I had in Afghanistan, that is why I can now solve my own problems and do something about my worries and thoughts myself.*” (P1).

^2^ “*[ …] My flatmate, when he has a small problem, he thinks the whole day is ruined. We [Syrians] are always relaxed. Everything is ok. [ …] When we have a big problem, we laugh because we always had problems in Syria.*” (P30).

### Behavioral coping strategies

We also identified behavioral strategies that manifest aspects of the process as well as the capacity for positive adaptation among our participants. These strategies include work as a distraction and source of meaning, withdrawal from stressors, connecting to cultural roots or faith, processing through creative outlets, and seeking mental health care.

Several participants described using **work** as a distraction from negative thoughts and problems (quotes 1–2 below). One participant also ascribed beneficial effects to the social pressure to regulate one’s mood at work (quote 2). Work can also help participants cope with limbo by giving uncertain times some meaning (quote 3).

^1^ “*Work was the best thing [to help cope with trauma] because I don’t have time [to ruminate] and I don’t remember things anymore. I am busy with other things.*” (P1).

^2^ “*Now that I work, I cannot say I feel bad today – you should always smile; it impacts the climate at work for everyone.*” (P31).

^3^ “*[Work makes me feel good] in that I think it means my life isn’t just passing by for nothing. As I said, I’ve been waiting for four years to find out what will happen to me [ …]. So in that respect, I am relieved that I am doing something positive and not just sitting around, waiting to see what terrible things will come my way.*” (P5).

One participant copes with the pressure of integration, especially difficulties with language, by **withdrawing** (quotes 1–2 below). The negative flip-side of this strategy is that she feels she is avoidant and choosing not to face problems (quote 2). It appears that she is also withdrawing more and more, becoming reliant on this coping mechanism:

^1^ “*I leave everything behind, [ …] stay home for like two days, then I can go back to something good to do. I have to take a break [ …].*” (P49).

^2^ “*I stay in my shell. [ …] I might find a way to manage things later, but I don’t want to think about them now. Kind of like escaping.*” (P49).

A few participants, such as the first quoted below, seek out environments that feel familiar and allow them to connect to **cultural roots or faith**. One participant spoke about how connecting to her culture, largely meaning her religion, inspired her to overcome her initial feelings of emptiness in Germany and gave her a sense of direction (quote 2). Another participant feels consoled by his faith (quote 3):

^1^ “*Sometimes I go to [a certain park] because it reminds me of my hometown. Sometimes I go to mosques, I feel at ease there.*” (P39).

^2^ “*The emptiness at the beginning – what helped me get rid of it is my culture. [ …] I have a culture that tells me to fill [the emptiness] with useful things and not with useless things. [ …] [My culture] gave me motivation, and the impression that it is a beginning for me and not an end*.” (P37).

^3^ “*Allah consoles me when something terrible happens to me.*” (P33).

**Processing troubling past experiences through creative formats** can provide comfort, as the first quote below shows. The second participant quoted reported that this practice also helps him compartmentalize by having dedicated time to process the past.

^1^ “*Drawing has become the most comforting thing for me, and the other thing is writing. I am writing now about the story of running away and my emigration.*” (P14).

^2^ “*I write, I spend almost half of the day writing. [ …] When I’m writing, I’m in the past, and when I’m not writing, I’m in the present.*” (P53).

Finally, several of our participants have sought **mental health care** and other forms of counselling to deal with past and present stressors. Some of their reflections on seeking this type of support, captured in the second and third quotes below, show that overcoming the stigma surrounding mental health and mental health care is often part of this process:

^1^ “*I actually also need help and have sought it. There’s this person, not a teacher, but a social worker, who I used to talk to. Especially in my first year [in Germany], I saw here twice a week then.*” (P45).

^2^ “*I didn’t really tell my family [that I went to see her]. I don’t think they would have thought it’s a good idea, that it’s not cool to seek help – what’s wrong with her? [ …*]” (P45).

^3^ “It’s *still something shameful for people to talk about. Even my own mother would be ashamed to tell others that I take an antidepressant. Not me. [ …] I tell everybody that I’ve been suffering from the last seven years, for not knowing where to go, what to do and [ …] yeah I had visited a therapist. [ …] Actually, it’s a selfish thing in a good way [to seek help for your mental health].*” (P48).

### Self-ascribed resilience as an enduring capacity

Some of our participants experience their strength in facing adversities as a trait or lasting characteristic rooted in their personality (quote 1), upbringing (quote 2), or positive past life experiences (quote 3). These self-assessments manifest an enduring or repeated capacity for positive adaptation in the face of adversity.

^1^ “*I am quiet and calm in general. Any situation I face doesn’t just make me sit whimpering in the corner, I go the steps, so I don’t get strong depression. I always get out of [bad] situations I enter. If I failed a language course, [ …] it’s no problem for me to register again.*” (P42).

^2^ “*I am calm and content, I would say. [ …]. [My mother] taught me always to be calm, always be happy. Always try to be satisfied with life no matter what is actually happening to you.*” (P46).

^3^ “*I don’t have a depression as many others here because I had a good past and that gives me something to hold on to.*” (P20).

### Volunteering, activism, and work for refugee causes

Several of our participants volunteer or work for refugee causes, employing their own experiences to become helpers to and advocates for others in similar situations. These activities appear to be a part of the process of positive adaptation. Activism and volunteering promote feelings of connection, agency, meaning and identity within participants who face isolation, long phases of unemployment, and the loss of roles that defined them in their pre-migration lives (quotes 1–2). One participant attributes the ease with which he felt he was able to integrate to the social networks that emerged from a large refugee protest (quote 3). Translating insights from hardships into helping others or even into political demands may also give these hardships meaning and provide a sense of distance from problems (quote 4):

^1^ “*Helping others also helps me – a lot! I feel like I am not alone, and while I can’t find work, I can do something for society, for myself, my family, not just sit around.*” (P52).

^2^ “*[Volunteering to teach] made me feel like when I was in Syria. I felt like I was regaining myself again since I’m a [ …] teacher and this is what I used to do for a living.*” (P3).

^3^ “*I was very lucky to [ …] to have joined the refugee protest [ …]. The social structures that emerged in this time are still there. That means that it was very, very easy for me to integrate.*” (P53).

^4^ “*So, the problems I have, now I have changed them into a vision of helping other people.*” (P34).

In many cases, volunteering, activism, and work for refugee causes also come across as manifesting the outcome of as well as the capacity for successful adaptation: many of the participants quoted here can help others because they have overcome some of the issues they see others facing (quotes 1–3 below). They demonstrate a sense of their rights and the confidence to make demands (quote 1), as well as a sense of having mastered specific aspects of integration such as navigating intercultural differences (quote 2). Some aim to use their language skills and their expertise on refugees’ needs to give voice to others (quote 3). Many of these participants conceive of themselves as communicators more generally, having understood problems and potential solutions and wishing to convey them to others in their community and to host country policymakers:

^1^ “*I am trying to do something for my [housing facility] because there are people who cannot even help themselves - they are so scared [ …]. But I am not scared anymore, because I know that [the housing facility leadership] cannot do anything [to me]. [ …] If I can speak, I will speak. [ …] I will get a [private apartment] afterwards. But I think there are people who need my help right now.*” (P34).

^2^ “*I would like to explain the differences between Syria and Germany to the Syrians. I would like to give seminars to the students in Syria. Or help them get scholarships, so they have the chance to explore the world. I would like to do many things.*” (P6).

^3^ “*I would do a much better job with being a social worker and interpreter, to help those people who can’t deliver their ideas. People who have the language block, that they want to express what is wrong with them.*” (P48).

Some of our highly proactive and emotionally stable participants involved in refugee causes were activists in their countries of origin. In other words, some of these individuals appear to have a long history of confronting adversity with proactive – and often courageous – involvement. They manifest a particularly pronounced capacity for positive adaptation. One such participant became active immediately upon arrival and gained increasing influence:

“*I arrived in Germany in [month, year], and in that [same] month, I organized a demonstration [ …]. The demands were related to basic life needs in Germany [ …]. On this basis, I started working with civil society and humanitarian organizations here in Germany. I also started the political work. [ …] The goal was to reach more realistic solutions and to make decisions with a stronger connection to people in life [ …].”* (P8).

## Factors facilitating successful adaptation

### Social support

Social support and new social connections in the host country in general, were instrumental in improving many participants’ mental health and helping them face challenges in Germany. Participants reported feeling a sense of acceptance (quote 1 below), security (quote 2), belonging (quotes 1 and 3), and being able to ward off isolation (quote 4) thanks to social connections, especially to Germans. They also benefit from very concrete support, e.g. with legal processes and finding housing. These experiences are in contrast with those of many participants who feel isolated and rejected in Germany.

^1^ “*I have my own contacts now. [ …] They accepted me and I accepted them. I got used to it. The feeling of being a stranger was gone. I started to see many people who are like me here. That has helped to adapt better.*” (P28).

^2^ “*We are more relaxed now. Especially since having met people who help us in all situations.*” (P32).

^3^ “*I play football with these German friends and I don’t feel like I’m the only Afghan there. We greet each other, eat together and play. It feels like a family.*” (P10).

^4^ “*Wherever I find gatherings for Germans or Syrian/Germans, I participate. I always put myself in the atmosphere, I don’t get isolated because I fear isolation, it brings me depression.*” *(P42).*

Many participants’ experiences also show that infrastructure dedicated to providing social support for refugees can, indeed, play a vital part in promoting well-being and feelings of acceptance. Our participants mentioned language tandems (quote 1 below), connections to families hosting refugees in their home (quote 2), refugee-centered social projects and meet-up events (quotes 3 and 4) as providing support on many levels. These resources provide practical and psychological support, boost confidence, comfort, sense of one’s rights, and motivation. Furthermore, language teachers (quote 5) are individual actors involved in the integration process who seem to be particularly impactful when they are supportive. They are the first Germans many refugees have sustained contact with and can provide a sense of familiarity and acceptance.

^1^ “*One of the important things that happened to me when I first arrived in Germany: I had a language tandem. She helped me get to know the city a lot. She took me to the cinema. We are still in contact until today. She was the first German person to invite me into her house and introduce me to her kids. All of that has given me more confidence in myself*.” (P6).

^2^ “*A friend of mine lives with a German family [ …]. When I first arrived, they welcomed me and protected me. The first months were so complicated, with so many appointments – they were always there. When it was my birthday, they had a surprise for me. [ …] It was like being at home.*” (P23).

^3^ “*There’s a regular event that I attend. It takes place in a church. [ …] The groups are mixed [migrants and locals] and there’s no difference between people. This improved my mood and pulled me out of my isolation. It improved my psychological problems.*” (P11).

^4^ “*We go to women’s associations and attend workshops. [ …] I feel that the events make us feel comfortable talking about what we want and what is needed and what we lack, and we find people who motivate us.*” (P22).

^5^ “*When I first arrived, a teacher took care of me so that I was not alone. She did so much for me. That’s why I am healthy now. They [two teachers] did everything for me. [ …]”* (P1).

Of course, having family members in Germany can be a major emotional resource (e.g. quote 1 below). Some of our participants (e.g. quote 2) also report leaning on their family members through long-distance communication.

^1^ “*When I feel longing, I go to visit my family. It makes me feel better instantly*.” (P6).

^2^ “*What gives you strength?*” (interviewer) “*That I talk to [my mother] on the phone every day.*” (P46).

### Experiencing migration as an opportunity for self-expression, belonging, and personal development

This theme identifies a factor facilitating successful adaptation based on a type our analyses revealed among our participants: those who a) are mentally well and appear to be integrating with relative ease and who b) also view migration as an opportunity for self-expression, belonging, and personal development. These participants were mainly highly educated men in their twenties and thirties. This attitude toward migration usually seemed to be a result of these individuals having felt unable to live in accordance with their values and identity in the country of origin. For example, one participant who comes across as remarkably well-adjusted was a persecuted oppositional activist in his country of origin. He suffered a loss of sense of belonging to his country and feels he can live more freely and more in accordance with his values in Germany:

“*Being in an internal exile is much worse than being in an external one,’ this is hard to translate, but what I mean is that I felt more like a stranger in Sudan and had a yearning to leave and not the other way around.*” (P53).

Another remarkably energetic participant values in Germany the individual freedom he missed in his country of origin and expressed enthusiasm about his relocation. He also attributed the ease with which he feels he is integrating in part to having been more “open” regarding other cultures and values compared to his peers in Syria. Through resettlement, he feels he has gained greater clarity of opinions, framing migration as an opportunity for personal growth:

^1^ “*It was an amazing opportunity in my life to have fled here. I was reborn here, but with memories. [ …] I’ve always had many opinions that I couldn’t show in Syria. I want to experience a lot and this wasn’t possible, and you always have to somehow – yes – lie. Things that I didn’t even agree with, but somehow I’m supposed to say ‘yes’. No. This time now is for me. I have the feeling that no one is watching me here. I am free.*” (P23).

^2^ “*I am very open and I was already very open in Syria, but I think I’ve improved, my character has improved, in Germany.*” (P23).

Even participants who express ambivalent emotions since arriving in Germany, such as the woman quoted here, derive positivity from their newfound freedom of expression:

“*I have mood swings. Sometimes so motivated and other times a bit let down. [ …] In general, I feel my positive energy is much higher in Germany [ …] Sometimes, I feel so motivated. This place makes me feel that I exist. I can freely express my opinion.*” (P24).

Others who seem to be content and active in Germany express being drawn to Europe culturally, feeling they belong, and having wanted to emigrate independent of their flight reasons (quotes 1–3 below). One of these participants (quote 1) emphasized the importance of religious and sexual freedom throughout his interview. As a new member of a religious community which is a minority in his country of origin, he feels more comfortable with Germans than with co-nationals. Another participant (quotes 2–3) feels that human life is inadequately valued in his country of origin. He can engage in an open discourse with people from various backgrounds in Germany and even prefers more individualist norms around socializing (quote 3). This is in direct contrast to many participants who suffer from missing the close-knit social network from their countries of origin and find German society to be socially cold.

^1^ “*My way of thinking is very Western. I feel like I belong here. I didn’t go to school for long, but I’ve seen a lot of the world. I’ve been to many countries. That’s why I think like a European.*” (P10).

^2^ “*I am very separated emotionally [from Syria]. I always felt I was going to leave this country, in a way or another. Yeah, like in my young brain, I always wanted to leave, if not to Germany then to somewhere else, anywhere else.*” (P48).

^3^ “*For me, I was never a social person. So I really feel happy in Germany, the society fits my standards.*” (P48).

An appreciation of the relatively greater diversity in Germany compared to their countries of origin is something many of the participants represented in this category – who, perhaps notably, all live in Berlin – share. In many of these cases, this is linked to having previous personal experiences of being an ‘other’ culturally or previous intercultural experiences. For example, a participant who has never lived where he is accepted as belonging (“I was born as a refugee. Being a refugee is nothing bad for me” (P30)) contrasts his own appreciation for diversity with others’ resistance to it:

“*Every person is [different] here. And that is beautiful. This dialogue between people here, I always learn something new. I really enjoy that. But many refugees don’t like this because of homesickness and wanting their own culture, their own things, their own pyjamas.*” (P30).

### Experiencing migration as an opportunity for women

While the above examples are interestingly mostly from very educated male participants in their twenties and thirties, a few of our very educated female participants in the same age range experience a gender-specific “coming into their own” in Germany. These participants mostly report mixed experiences in terms of their well-being in Germany, but they show a sense of elation at perceived newfound freedom from gender role restrictions.

One woman feels invigorated by a sense of opportunity for herself as a mother in her thirties in Germany and experiences a more emancipated relationship with her husband post-migration. She feels that she is among those within the refugee community who have “found themselves” by breaking from old customs that limited them:

^1^ “*I feel I’m younger [in Germany]. I do not know why. You feel that they [in Syrian society] have determined your task of procreation and your task is limited to cooking and home. [ …] Your purpose in life ends in the early 30s. I look at the older people [here] who wear pink and dye their hair pink, even though they are old. They still have a love for life. I feel that I am at the beginning of my life, and I want to do many things. This is a thing that has changed.*” (P14).

^2^ “*My relationship with my husband changed. Now I can say ‘no’ without justification. [ …] [My husband] became quieter, more polite, and I feel that he has been adjusting to the atmosphere of the society here and the changes in his wife. Thank God!*” (P14).

^3^ “*There are people who [ …] have no desire for rebellion or change, but there is a large group that felt that they found themselves here. Like they lost a connection and found it in a certain place. For me, yes.*” (P14).

A woman who says she feels at home in Germany and has recovered her mental health after extremely harrowing experiences emphasized that she never felt comfortable in her country of origin because of her restrictions as a woman. After resettlement, she feels she can ‘pursue her dreams’ of studying and moving through the world independently:

^1^ “*When I was much younger, I used to be outside all the time, but when I grew older, then it was the time that restriction starts - girls should not go outside and so on. [ …] So, it’s not like here in Germany - you can do whatever you want. There are no restrictions, but in Pakistan, in my situation, I cannot remember anything that was good.*” (P34).

^2^ “*Do you sometimes feel torn between your home country and Germany?*” (interviewer) “*No. I feel like home here.*” (P34).

Other female participants, such as the first two quoted below, take pride in having greater professional and educational freedom and newfound independence since resettling. The final quote below shows that a more symbolic freedom can also have an impact on well-being.

^1^ “*What are my feelings? Maybe that I am proud of everything that I have been able to do here. [ …] Back home, it’s not common or not positive for a woman to work as a waitress. [ …] And my dream as a child was really to become a waitress. And now I’ve fulfilled my dream.*” (P51).

^2^ “*Now [ …] I am allowed to do what I want and what I feel like. It was not like that in my family. I always had to ask whether I was allowed to do something. [ …] My mother chose my major in Damascus.*” (P51) “*But now you chose?*” (interviewer) “*Yes*.” (P51).

^3^ “*Riding a bicycle is something I didn’t do in Syria. When I ride it, I feel freedom and happiness. In Syria, it wasn’t common for a girl to ride a bike. It really makes me feel happy.*” (P6).

### Being a parent

Several participants who are parents presented a positive attitude toward their new lives in Germany that is based on their children doing well, as the quote below shows. The educational opportunities afforded to their children in Germany were often mentioned as a benefit of having relocated, helping these parents think more positively about their forced migration.

“*[Feelings of comfort] come from my kids, always. They have a very normal life and they get everything they need. They live in safety and stability, which is the most important thing. That is enough for me to feel at ease.*” (P28).

Some parents fight mental health problems and make an effort to reduce stress and negativity for their children’s sake. They have their children in mind when dealing with everything from past traumatic experiences to feeling overwhelmed, without direction, or depressed in the host country (quotes 1–3). Sometimes this comes in the form of suppressing problems and experiencing pressure as a consequence (quote 3):

^1^ “*So I have a lot of frustration and feel that I don’t want to do anything, sometimes I wish I could die because I am so tired from all of this. But then I still keep remembering my son, [ …] so I should leave all these negative feelings and just look on the bright side.*” (P29).

^2^ “*We always try to be in good spirits for our child*.” (P25).

^3^ “*But I have to be strong and fight back. Not just for myself, also for the sake of my kids. At the same time, this puts too much pressure on me. I can’t even express my sadness and anger so that it won’t have a negative impact on my kids. Sometimes I lose control, and I can’t help it.*” (P3).

For a few participants, a focus on their children is a way to counterbalance their own emotional struggles. On the one hand, this attitude is a source of solace; on the other hand, it may result in these participants abandoning hopes for their own development after resettlement:

^1^ “*I am happy but at the same time depressed. Happy to see my children happy but depressed for myself because I am sitting at home and doing nothing.*” (P17).

^2^ “*It is all about my children. [ …] We have no dreams anymore.*” (P17).

While many of our participants, including parents, reported suffering from a sense of meaninglessness while their lives feel on hold as a result of various uncertainties and restrictions, some parents are able to focus on the things they do for their children as tangible, meaningful activities:

“*I’m not doing any activities for myself. But I’m doing them for my son. Like swimming and music, etc. This makes me feel like I’m making a real-life investment. Which is my son.*” (P24).

In two participants who had children post-migration, the birth of their child made them feel more like they were experiencing a new beginning (quotes 1–2 below). In one of these cases (second quote), the birth of this child appears to have helped the parents heal from the traumatic loss of their first child and the harrowing flight journey surrounding it:

^1^ “*My life in Syria is over. I’m starting a new life in here. Especially now that I have a son.*” (P24).

^2^ “*But it was all the luck of my daughter, that we had afterwards. [ …] After she was born, she is so lucky – everywhere we’ve gone, everyone is happy and we never had any problem after she was born.*” (P34).

### Being young

Some – though not all – of our very young (under 24 years old) participants appear to be doing particularly well in Germany by virtue of factors related to their youth. These participants, all well-educated, seem to have adjusted quickly and feel confident in their futures, able to focus on opportunities. Comparing their attitudes with the reflections of older participants, it appears that they are able to move forward in part due to not having experienced a perceived loss of status and future prospects due to migration. A youthful trust in a long future full of opportunities comes through in some very young participants’ interviews:

“*I mean, you have your whole life ahead of you and can do so many wonderful things and study or work or start a family and get married – I don’t know, just live life.*” (P13).

Relatedly, even though some did seek mental health care, some of these young participants also seem to let go of difficult past experiences more quickly than older participants:

“*Of course I experienced difficult things, flight and war, I don’t know. But now that it’s over, I’ve already forgotten everything. I started from scratch and I’m happy with everything I’ve already achieved up until now.*” (P50).

These particularly well-adapted young participants also tend to have fewer responsibilities in the integration process. With one exception, they came to Germany accompanied or preceded by supportive and capable parents. Finding their place amongst their peers at school appears to be one of our youngest participants’ main integration struggle. From the way these young participants describe this struggle, it appears that this is not simply their focus because they have reduced responsibility: it also seems like their age-appropriate preoccupation with their peer group may focus their worlds in a protective way. For the two quoted here to illustrate this point, changing to a more academically oriented school and changing from a small-town school to a city school, respectively, made the difference:

^1^ “*Since having friends, [ …] I also have a boyfriend [ …] and my best friend now also lives in Germany, and since then I’ve been happy. I actually feel totally ok. Yes*.” (P50).

^2^ “*But then, when school started, and I found friends, the worries were suddenly gone and now everything is going well.*” (P13).

Doing well in terms of academic achievement is another primary concern and a source of satisfaction and motivation. Unlike their older counterparts, many of whom feel directionless, very young refugees who are in education may benefit from straightforward metrics of progress:

“*When I do something well, when I achieve something at school or in the traineeship, that makes me feel content.*” (P50).

Reflecting on differences in well-being between herself and her mother, one participant said she thinks it is easier for younger people to integrate, make new contacts, and learn the language – in part because of their greater flexibility, in part because educational facilities enable integration and language learning more readily than courses for adults:

^1^ “*My mom also has these phases when she is depressed, which I can understand. I mean, older people cannot integrate as quickly, and she has no friends, only her family.*” (P45).

^b^“*It’s probably completely different for me than for my parents, life in Germany. I am only 19 years old, my experiences are completely different.*” (P45).

## Discussion

Based on a qualitative analysis on different aspects of resilience among adult refugees in Germany, this study presents a range of manifestations of the process of, capacity for, and outcome of successful adaptation as well as factors facilitating positive adaptation.

### Manifestations of the process of, capacity for, and outcome of successful adaptation

Our participants use specific cognitive coping strategies and ways of framing circumstances in order to maintain their functioning and mental well-being despite adversities. Several of these strategies have been identified in other studies as well, for example, acceptance and focus on the present or future [26], belief in one’s own inner strength [26], which is comparable our “internal locus of control” category, and favorable comparisons to others in the same situation [25]. Favorable comparisons between the receiving country and the country of origin have also been found as related to resilience in other analyses [26, 29]. The strategy of active forgetting, also identified in previous studies on refugees [39], raises the point that coping can be active or avoidant and adaptive or maladaptive [40]. Active forgetting is an example of an avoidant coping strategy. As such, it may be functional and thus conducive to positive adaptation as a short-term but not necessarily a long-term solution [41].

Among the behavioral coping strategies our participants use to manage the mental health repercussions of the stressors they face, there are also avoidant strategies. Withdrawal into the private realm is an avoidant strategy and may have ambivalent effects, as the participant who reports this behavior openly discussed. Experiencing work explicitly as a distraction from worries also comes across as potentially ambivalent in our participants, unlike using it as a way of bringing meaning to a limbo state. Processing experiences using creative outlets as well as connecting to cultural roots and faith are active and likely adaptive forms of coping. While spirituality and faith are major themes in many qualitative studies on refugee resilience [9], they were not strongly represented in our interviews. Perhaps our sample happened to be less religious, or perhaps it takes more specific questions, including specific questions about coping strategies, to elicit mentions of faith.

Seeking mental health care is not often framed as a coping method in the literature (except in [31]); however, like the other examples presented in this theme, it represents a behavior actively undertaken to manage stress responses to adverse experiences. Our participants also display inner strength in seeking mental health care because it often means overcoming stigma around mental health treatment, which may be pronounced among refugee communities  [27,42].

Some participants’ impression that they have something like an enduring capacity for successful adaptation due to different factors including life-long characteristics, an attitude instilled by a parent, or a good past in part chimes with the classical understanding of resilience as a trait (e.g. [43]). This view has been critiqued for implying a binary between those who can and those who cannot overcome adversity [44]. However, the existence of something like an enduring or repeated capacity for positive adaptation in the face of adversity based on personality factors and particular resources seems highly likely. In any case, it is noteworthy that some of our participants perceive themselves as uniquely robust. The question arises whether this self-image might itself act as a cognitive strategy for positive adaptation.

Volunteering, activism, and work for refugee causes manifest as a part of the process of adaptation. In a mechanism similar to what has been described in the literature as ‘altruism born of suffering’ [45] and ‘adversity-activated development’ [5], some participants translate their adverse experiences into activism or helping others. In line with the classic clinical perspective on this phenomenon, some of our participants seem to employ their volunteering or activism almost as a coping mechanism that helps them find meaning in their suffering [45] and build agency by avoiding the role of victim [46]. Indeed, volunteering has been shown to elicit a range of beneficial outcomes for refugees, including feelings of self-fulfillment and sense of belonging and overall improvement of mental health [47], as some of our participants described.

Volunteering and activism are also manifestations of the capacity for and outcome of positive adaptation. Our participants presented in this theme are in part able to help because they have overcome or are overcoming. They use resources such as language abilities, cultural knowledge, and knowledge of their rights in order to help others. Some individuals in our sample were already activists in their countries of origin and then became active for causes in Germany. This again suggests the possibility of an enduring or repeated capacity for, in this case, a certain type of adaptation: responding to adversities with remarkable proactivity. Two characteristics linked to resilience as a trait in the literature that would appear to be a prerequisite for turning suffering into action include high energy levels and the ability to detach and conceptualize problems [43].

While manifold links between volunteering and activism and resilience can be found, a note of caution is warranted. Any normative appeals that refugees should become volunteers or activists disregard the many reasons why someone may be unable, reproduces the prejudice of the “lazy refugee” [48], of which our participants are painfully aware, and potentially adds to a pressure to perform as “good refugees” [49]. It also promotes unpaid or underpaid forms of labor in a community that is already economically disadvantaged.

### Factors facilitating successful adaptation

Social support is a primary theme in most studies on refugee resilience [9]. Our findings resonate with summaries from previous studies stating that social networks provide emotional as well as informational support and promote a sense of belonging [30]. The importance of infrastructure designed to provide social support, such as meet-ups and tandems, and key contacts in the integration process, such as language teachers, also comes across in our data.

We identified another potential protective factor from a pattern that emerged in our analyses: Several participants who appeared to be emotionally well and very active – i.e. those who came across as having adapted well to difficult circumstances – also exhibited a distinct attitude toward their relocation. They reported having been critical of, persecuted, or othered by, or simply having felt alienated and restricted by the dominant culture or governments in their countries of origin. After resettlement, they  feel that they are experiencing increased opportunities for self-expression and even a greater sense of belonging. These individuals contrast many of the other participants in our sample who may appreciate the safety and civil liberties they have gained through migration but feel deeply rooted in their societies of origin.

The observed pattern may be due to several underlying mechanisms. We have categorized it as a factor promoting adaptation under the assumption that positive attitudes toward relocation, which often appear to have predated resettlement, may justify struggles and provide determination and motivation. Conversely, better mental health and a more active life may also lead to more positive, empowered appraisals of migration – or these appraisals may function as strategies for overcoming struggles. There might also be a confounding factor underlying this pattern: Perhaps those who have enduring capacities or greater resources for successful adaptation are also the ones who questioned the social order in their countries of origin or who value experiencing new environments. For example, the high level of education among these participants may explain both positive adaptation and positive attitudes toward migration. Because multiculturalism is among the things these participants appreciate about Germany, it should be noted that all participants represented in this theme live in Berlin. This city prides itself in its multiculturalism [50]. It is very likely that circumstances in the receiving country impact how refugees' attitudes toward their resettlement develop, even those that appear to originate from pre-migration times.

Interestingly, the examples of this pattern in our sample are overwhelmingly male, perhaps owing to greater opportunity for being oppositional in the country of origin, more personal choice in the matter of migration, and greater opportunities in the host country [51].

However, some women in our sample reported a post-migration appreciation of newfound gender role freedoms in the receiving country that seems to be a source of well-being. A similar finding was presented in Liu and colleagues’ [11] qualitative resilience analysis. While refugee women are more likely to suffer from mental health problems in the receiving country than their male counterparts (e.g. [52, 53]), migration can increase autonomy, self-esteem, and social standing and provide new opportunities, as also summarized in a previous report [54]. However, several factors such as socioeconomic characteristics and the immediate home environment in the receiving country impact whether migration opportunities can level or outweigh the risks of migration for women [54]. Indeed, women in our sample who draw strength from newfound gender role freedom were mainly highly educated and young.

Although migration holds particular challenges for parents, such as having to manage childrearing alongside integration and facing acculturation-related intergenerational conflicts [55], we found that children can promote positive adaptation in refugee parents. In accordance with previous studies, we found that children can be primary sources of motivation to overcome difficulties, sense of meaning, and justification for sacrifices and hardships for their parents [55]. The experiences of the mothers in our sample also resonate with previous reports of refugee mothers’ well-being as strongly linked to their children’s adaptation process [56], which means their children may represent a source of hope independent of their own struggles. On the flip side, parents may also experience a double burden when their children are not doing well [56]. A focus on the children may come at the expense of parents’ willingness to invest in their own development post-migration, as we found in some of our participants, as well as participant quotations from other studies: “‘I have no expectations. [ …] I don’t care anymore. [ …] I just hope for a better future for my children” ([57], p. 316).

Our finding that being young seems to confer resilience in some cases is in line with an EU-wide study reporting that younger refugees experience an easier adaptation process than their older counterparts across the countries examined [57]. Our interviews suggest that young people with relatively good starting conditions, such as having migrated with family and being well-educated, exhibit a certain youthful hopefulness and flexibility. A youthful preoccupation with the peer environment and academic achievements may also be adaptive. An external advantage the youngest adult refugees often have is entering into educational facilities in the receiving countries, usually secondary schools. Schools facilitate the process of integration by providing social support and giving young refugees a sense of agency based on daily tasks to accomplish [58]. In line with our participants’ experiences, however, the school environment’s quality with regard to inclusivity is key to fostering social contact building, a sense of belonging, and a positive attitude toward education [59, 60].

We also observed that our youngest participants were not burdened by the feeling of having to “start from scratch” that was central to the experiences of many older participants. Because they were still in the process of completing their education before resettlement, our youngest participants have, indeed, lost less progress in their lives through migration. Many young refugees are focused on education and the desire to build a meaningful life in the receiving country, as has been observed previously [61]. Again, a major caveat here is that our young participants are all in relatively privileged positions concerning their domestic and educational situations.

### Limitations

A limitation of this study is that the interview topic guide featured several questions focused on stressors and mental health problems, which may have curtailed participants’ reflections related to strength and resilience. Furthermore, our reliance on native speaker Arabic and Farsi interviewers and translators for many interviews enabled participants to describe their experiences fluently; however, despite quality checks, it also means that the transcripts we used in our analyses may lack linguistic precision. It should also be noted that while we were able to recruit hard-to-reach participants with very limited educational backgrounds, our sample was on the whole highly educated, with over half having attended or completed university – a far higher proportion than in the population of refugees in Germany at large [3]. 19 participants were able to participate in German, indicating good integration along with one key parameter. While the level of education is mentioned as a factor in themes where it seemed to be particularly relevant, these are caveats regarding our results’ generalizability. On the content level, it is important to note that sorting reported human experiences into different resilience categories is not a straightforward process. Various manifestations of and factors promoting resilience are likely to function in various ways and to be highly interconnected.

## Conclusions

By identifying a range of manifestations of the process of, capacity for, and outcome of successful adaptation as well as factors facilitating successful adaptation in the face of adversity among adult refugees, this study contributes to a growing body of knowledge on resilience in this population. These findings may support clinicians working with refugees by making them aware of potential strategies and sources of strength specific to this client group. They also contribute to moving the academic discourse and potentially clinical practice toward a more strengths-based view on refugee mental health and integration processes.

## Supplementary Information

**Additional file 1.**

## Data Availability

To protect the identity of the participants, the data used in this study is not available to third parties.
