# Supplementary material for: A qualitative study on resilience in adult refugees in Germany
Source: BMC Public Health. 2021 Apr 30;21:828. doi: 10.1186/s12889-021-10817-6 (PMC8086291; doi:10.1186/s12889-021-10817-6)
Supplement: Supplementary file 1 — Additional file 1. [file 12889_2021_10817_MOESM1_ESM.pdf]

APPENDIX

A Qualitative Study on Resilience in Adult Refugees in Germany

Lena Walther[1]*, Julia Amann[1], Uwe Flick[2], Thi Minh Tam Ta[1], Malek Bajbouj[1], Eric Hahn[1]

[1]Department of Psychiatry and Psychotherapy, Charité University Medicine Berlin, Berlin, Germany

[2]Department of Education and Psychology, Freie Universität Berlin, Berlin, Germany

*** Correspondence:**
Lena Walther
lena.walther@charite.de

Contents:

Study Flyer Text – pg 2
Topic Guide – pg 3-5

Study Flyer Text

We want to hear from you: How are you doing in Germany? What are you experiencing in your new environment?

What?
We are researchers from the Free University Berlin and the Charité University Hospital Berlin. We would like to invite you to have a conversation with us:

- in German, English, Arabic, or Farsi/Dari
- about 1 hour long
- just between you and one of us
- topics: How are you experiencing cultural differences or similarities in Germany? How are you doing emotionally? What do you like about life here, and what could be improved?

Where?

- Wherever you feel comfortable
- We are happy to invite you for coffee/tea/cake in a café!
- We will reimburse travel expenses

Why?

- Your story can help create better conditions for refugees in Germany: through our research, we want to understand what Germany and other countries need to know to offer arriving migrants the best possible conditions.

Are there disadvantages?
No!

- Your data will be anonymized immediately after our conversation and used only for research purposes. Your identity will not be revealed to anyone.
- You can end your participation at any time.

Topic Guide

Short Introductory Section:

- Country of origin?
- When did you arrive in Germany?
- How old were you when you left your home country?
- Did you choose to come to Germany specifically? Why?
- Do you wish to stay in Germany?
- What is your current legal status?
- What type of housing do you live in?
- Why did you flee?

Belonging and Values and Attitudes:

1) What is most important to you in life? What do you value most? (*Try thinking about basic topics, like security, family, faith or freedom)*

2) What do you like most about Germany and the way of living here? (*On the contrary, what do you like less*)

3) How do you feel when interacting with Germans? Do you notice any cultural barriers? **(*added after first interviews*)**

4) Does living here match the image you had of Germany before coming here? What is different?

5) Do you feel accepted in German society? Please explain why you do/don't.

6) When you think back to your life in XXXX, what did you like most about the society and culture? (*What gave you stability, what empowered, structure of society*)

7) Do you sometimes feel torn between your home country and Germany?
-> (*And what makes you feel this way - what is missing?)*

8) What activities do you do here in Germany that give you a feeling of comfort and familiarity? (*Which group / custom / activity gives you a feeling of familiarity at the moment*?)

<u>**Follow-ups -> As guideline for the interviewer to "go in deeper"**</u>

Political Ideals 1: How do you feel about the political system in Germany (*compared to the one in your home country*)?

Political Ideals 2: How would you change the political system if your vote alone could?

Gender 1: How would you describe your own ideals of a family? Has your relationship to your family changed?

Gender 2: How do you perceive the relations between men and women in Germany?

Religion: How do you perceive the meaning of religion in public life in Germany?

Well-being and arriving in Germany:

1) Which feelings dominate your daily life since your arrival in Germany?
Possible follow-ups:
- Could give examples like: calm, content, happy, excited, stressed, sad, angry, worried, anxious, irritated, generally emotionally well or less so?

2) What has made you feel this way?

3) How have these areas of life made you feel in Germany? (*those not yet addressed in Q 1 and 2, but please go through them one by one*)
   - social life
   - work life or education
   - housing situation
   - legal and bureaucratic matters
   - the new cultural environment

3) What currently burdens you the most? / What do you currently worry about the most?

4) Have your feelings changed and shifted during your time in Germany? How and why?

5) What do you think other people who fled from the same country as you have predominantly felt like since arriving in Germany – the same as you, or different?

6) How do your feelings and emotions influence the process of building a new life in Germany?
Possible follow-ups:
- How do your feelings influence activities like learning German, making new contacts here, looking for things to do, etc.? Do you ever have too many worries to do those things, for example?
- If not (no problems), where does this resilience come from?

7) Do you think that the current situation in your home country or your experiences here in Germany have a bigger impact on how you feel these days?

8) Do you think that the past or the present has a stronger impact on how you feel these days?

9) Do you think refugees in general face mental health challenges/ emotional stress? Why yes/no?
(*added after first interviews*)

10) Do you think women or men face greater stress during the process of migration and resettlement? Why?
(*added after first interviews*)

11) Do you think there is help for refugees who struggle with their mental health in Germany?
(*added after first interviews*)

12) Do you think stigma around mental health makes it hard for many refugees to seek support from friends, family and care providers here in Germany?
(*added after first interviews*)

13) How is mental health viewed in your country of origin?
(*added after first interviews*)

14) What would need to happen for you to be as happy, content and relaxed as possible here in Germany?
